# Jak3, STAT3, and STAT5 inhibit expression of miR-22, a novel tumor suppressor microRNA, in cutaneous T-Cell lymphoma

**DOI:** 10.18632/oncotarget.4111

**Published:** 2015-05-12

**Authors:** Nina A. Sibbesen, Katharina L. Kopp, Ivan V. Litvinov, Lars Jønson, Andreas Willerslev-Olsen, Simon Fredholm, David L. Petersen, Claudia Nastasi, Thorbjørn Krejsgaard, Lise M. Lindahl, Robert Gniadecki, Nigel P. Mongan, Denis Sasseville, Mariusz A. Wasik, Lars Iversen, Charlotte M. Bonefeld, Carsten Geisler, Anders Woetmann, Niels Odum

**Affiliations:** ^1^ Department of Immunology and Microbiology, University of Copenhagen, Copenhagen, Denmark; ^2^ Division of Dermatology, McGill University Health Centre, Montréal, Quebec, Canada; ^3^ Department of Molecular Medicine, Copenhagen University Hospital (Rigshospitalet), Copenhagen, Denmark; ^4^ Department of Dermatology, Aarhus University Hospital, Skejby, Aarhus, Denmark; ^5^ Department of Dermatology, Copenhagen University Hospital, Bispebjerg, Copenhagen, Denmark; ^6^ Faculty of Medicine and Health Science, School of Veterinary Medicine and Science, University of Nottingham, Loughborough, United Kingdom; ^7^ Department of Pathology and Laboratory Medicine, University of Pennsylvania, Philadelphia, PA, USA

**Keywords:** miR-22, cutaneous T-cell lymphoma (CTCL), mycosis fungoides (MF), STAT3, STAT5, JAK3

## Abstract

Aberrant activation of Janus kinase-3 (Jak3) and its key down-stream effectors, Signal Transducer and Activator of Transcription-3 (STAT3) and STAT5, is a key feature of malignant transformation in cutaneous T-cell lymphoma (CTCL). However, it remains only partially understood how Jak3/STAT activation promotes lymphomagenesis. Recently, non-coding microRNAs (miRNAs) have been implicated in the pathogenesis of this malignancy. Here, we show that (i) malignant T cells display a decreased expression of a tumor suppressor miRNA, miR-22, when compared to non-malignant T cells, (ii) STAT5 binds the promoter of the miR-22 host gene, and (iii) inhibition of Jak3, STAT3, and STAT5 triggers increased expression of pri-miR-22 and miR-22. Curcumin, a nutrient with anti-Jak3 activity and histone deacetylase inhibitors (HDACi) also trigger increased expression of pri-miR-22 and miR-22. Transfection of malignant T cells with recombinant miR-22 inhibits the expression of validated miR-22 targets including NCoA1, a transcriptional co-activator in others cancers, as well as HDAC6, MAX, MYCBP, PTEN, and CDK2, which have all been implicated in CTCL pathogenesis. In conclusion, we provide the first evidence that de-regulated Jak3/STAT3/STAT5 signalling in CTCL cells represses the expression of the gene encoding miR-22, a novel tumor suppressor miRNA.

## INTRODUCTION

Cutaneous T-Cell lymphoma (CTCL) is characterized by the proliferation of malignant T cells in a chronic inflammatory microenvironment. The etiology of CTCL remains only partially understood and, unfortunately, it has not been possible to identify one unifying genetic event or one particular oncogene of central importance for the malignant transformation and cancer progression. Instead, deregulation of signaling pathways including Signal Transducers and Activators of Transcription (STAT), src kinases, c-Myc, COX-2, NFκB, GATA3, TOX, and embryonic stem cell regulators appears to play an important role in the pathogenesis [1-11]. In particular, the Interleukin-2 receptor common gamma chain (IL-2Rgc), the associated Janus kinase-3 (Jak3), and the down-stream effectors (STAT3 and STAT5) have attracted substantial interest in this regard. Indeed, the IL-2Rgc-signaling cytokines IL-2, IL-4, IL-7, IL-15, and IL-21 are implicated in early pathogenesis whereas constitutive, interleukin-independent activation of the Jak3/STAT3 pathway is believed to play a key role in progressive and advanced disease [12-15]. The aberrant activation of the Jak3/STAT pathway and interleukin-independent proliferation of malignant T cells appears to be in part a result of deficient expression and/or function of negative regulators such as Suppressors Of Cytokine Signaling-3 (SOCS3) and the protein tyrosine phosphatases (e.g. SHP1) [16-18]. Importantly, the Jak3/STAT pathway (i) promotes the expression of IL-5, IL-10, IL-17A, IL-17F cytokines, (ii) regulates the production of angiogenetic factors, and (iii) confers resistance to treatment with HDAC inhibitors in malignant T cells [19, 7, 15, 20-24]. Yet, relatively little is known about STAT target genes in CTCL and how STAT3, STAT5, and their co-activators and/or co-repressors regulate of expression of disease-associated genes.

Recently, a new class of post-transcriptional regulators, non-coding microRNAs (miRNAs), has received considerable attention in relation to CTCL. Skin lesions from CTCL patients displayed a distinct miRNA expression signature. Furthermore, a minimal miRNA classifier consisting of only three miRNAs (miR-155, miR-203, and miR-205) was able to distinguish between malignant and benign dermatoses with high accuracy suggesting that miRNAs profiling is a powerful diagnostic tool in CTCL [25-27]. Importantly, expression of several miRNAs including miR-155 and miR-21 were associated with progressive disease and a poor prognosis [27-31]. miR-21 confers resistance to apoptosis, while miR-155 promotes malignant proliferation [14, 29]. Conversely, the expression of other miRNAs is often lost in CTCL skin lesions when compared to benign dermatoses and normal skin [25, 28, 30]. Some of these miRNAs are now recognized to act as tumor suppressors as documented by their ability to inhibit tumor growth and/or metastasis [32, 33]. mir-22 expression is down-regulated in a number of cancers and has been assigned a role of tumor suppressor miRNA in colon and breast cancers [33-37]. In a recent study of miRNA expression in Sézary syndrome, a leukemic variant of CTCL, Ballabio et al. described a loss of miR-22 expression in circulating CD4+ T cells [38].

It is largely unknown what promotes the expression of onco-miRNAs and repression of tumor-suppressor miRNAs in CTCL. In the current work, we demonstrate that malignant T cells display down-regulation of miR-22 expression and provide mechanistic evidence that Jak3/STAT signaling inhibits the expression of this gene. This, in turn, contributes to elevated expression of MYCBP and MAX (co-activators of the c-Myc oncogene), and NCoA1, a transcriptional regulator previously associated with neoplastic transformation events [34]. Our work provides evidence that aberrant Jak3/STAT activation represses a novel tumor suppressor miRNA in CTCL.

## RESULTS

### Loss of miR-22 expression in malignant T cells

**Figure 1:**
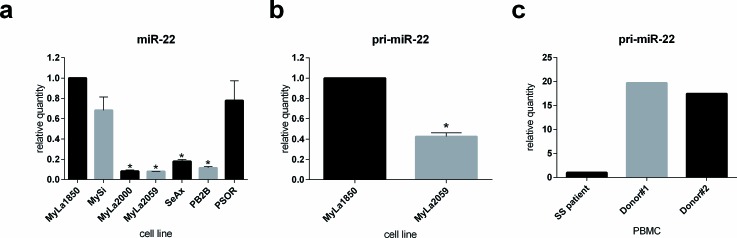
Expression of mature and primary miR-22 in CTCL as determined by qPCR **a.** miR-22 expression in non-malignant (MyLa1850, MySi) and malignant (MyLa2000, MyLa2059, SeAx, PB2B) CTCL T cell lines as well as one T cell line derived from psoriasis vulgaris patient (PSOR), reference U6, *n* = 3. **b.** primary miR-22 (pri-miR-22) expression in non-malignant (MyLa1850) and malignant (MyLa2059) CTCL T-cell line. Reference GAPDH, *n* = 3 **c.** pri-miR-22 expression in primary Peripheral Blood Mononuclear Cells (PBMCs) derived from two healthy donors relative to one patient diagnosed with Sézary Syndrome, reference GAPDH.

Decreased expression of miR-22 was recently reported in several solid cancers and in circulating CD4^+^ T cells in Sézary syndrome [35-39]. As shown in Figure 1A, miR-22 expression was significantly downregulated in malignant CTCL T cell lines (MyLa2000, MyLa2059, SeAx, and PB2B) when compared to non-malignant (i.e. reactive) T cell lines obtained from CTCL skin lesions (MyLa1850 and MySi) and psoriatic skin (PSOR). Notably, the expression of pri-miR-22 was also decreased in the malignant T cell line MyLa2059 when compared to non-malignant T- cell line (Figure 1B). Similarly in Sézary syndrome peripheral mononuclear cells miR-22 expression was decreased when compared to the cells from healthy volunteers (Figure 1C).

### Jak3/STAT signaling represses miR-22 expression

**Figure 2:**
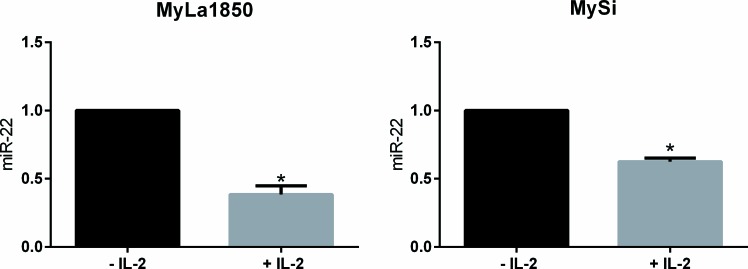
Effect of the T cell growth factor, IL-2, on miR-22 expression Expression of miR-22 in IL-2 sensitive, non-malignant, CTCL T cells (MyLa1850 and MySi). Cells were depleted of IL-2 for 48 hours (– IL-2) or depleted of IL-2 for 24 hours, followed by 24 hours of IL-2 supplementation (+ IL-2). miR-22 expression was determined by qPCR using U6 as a reference *n* = 3.

**Figure 3:**
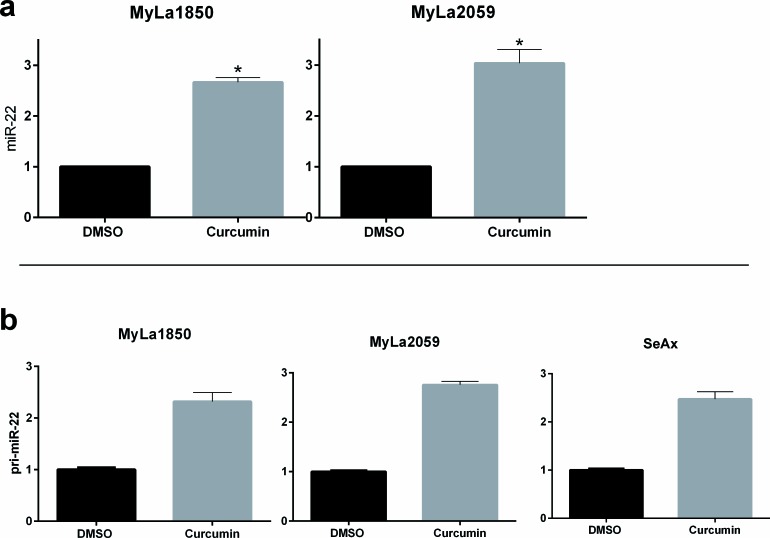
Curcumin treatment increases expression of mature and primary miR-22 miR-22 **a.** and pri-miR-22 **b.** expression measured by qPCR in non-malignant (MyLa1850) and malignant (MyLa2059, SeAx) CTCL T cells subjected to 24h treatment with 20μM curcumin or DMSO (control).**a.** Reference U6, *n* = 2. **b.** Reference GAPDH, error bars reflect variation in technical triplicates.

**Figure 4:**
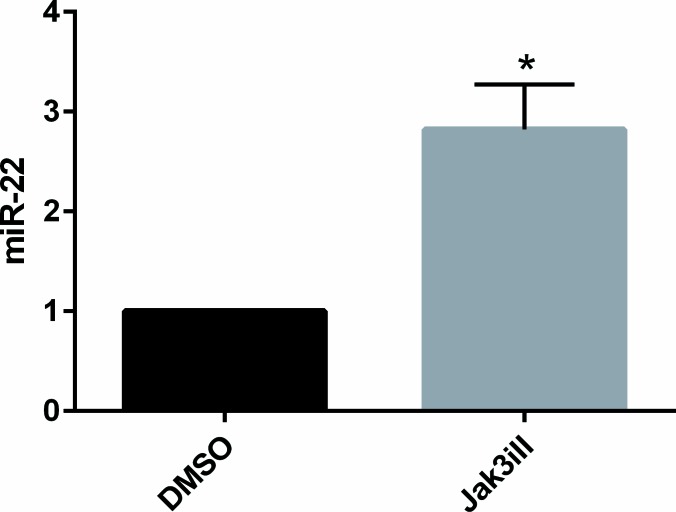
Inhibition of JAK3 increases expression of mature miR-22 in malignant CTCL cell line MyLa2059 miR-22 expression in MyLa2059 following 24 hours treatment with Jak3iII (40ug/mL) or DMSO control. Measured by qPCR, reference U6, *n* = 3.

**Figure 5:**
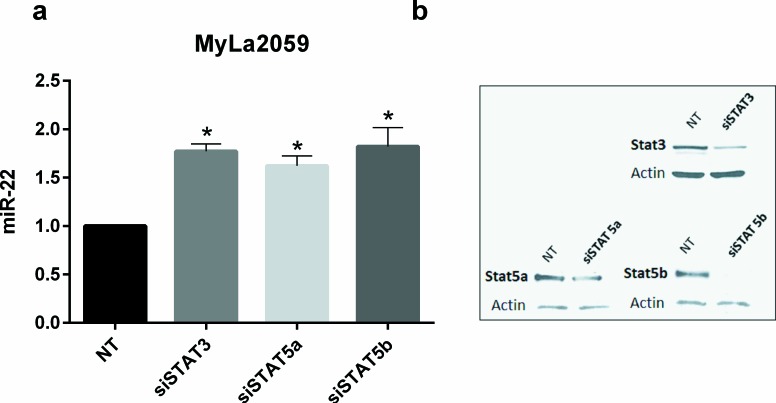
Transient knockdown of STAT3 and STAT5 genes increases expression of mature miR-22 in malignant CTCL cell line, Myla2059 **a.** miR-22 expression in MyLa2059 48h following transient transfection with siSTAT3, siSTAT5a, siSTAT5b or non-target (NT) control. Reference U6, *n* = 3, error bars reflect variation in technical triplicates. **b.** Representative Western Blot showing knockdown efficiency of siRNA transfections, 48h.

Il-2Rg-signaling cytokines regulate expression of multiple miRNAs through the Jak/STAT pathway. As shown in Figure 2, IL-2 induced a significant decrease in miR-22 expression in non-malignant T cell lines MyLa1850 (Figure 2, left panel) and MySi (Figure 2, right panel). Conversely, inhibition of IL-2R signaling by curcumin (a broad-range Janus kinase inhibitor) triggered in IL-2 treated non-malignant T cells an increased miR-22 expression when compared to the vehicle control (Figure 3A, left). Likewise, in malignant T cells that are known display a constitutive, aberrant Jak3 activation [40], curcumin produced an up-regulation of miR-22 (Figure 3A, right). Notably, curcumin also enhanced pri-miR-22 expression in malignant MyLa2059 and SeAx T cells (Figure 3B, right and central panels) and in IL-2-treated non-malignant T cells (Figure 3B, left panel). Since curcumin inhibits other kinases in addition to Jak3 in malignant T cells, we tested the effect of a more selective Jak inhibitor, Jak3- inhibitor II, on miR-22 expression in malignant T cells. As shown in Figure 4, Jak3- inhibitor II triggered an increase in miR-22 expression comparable to the effect of curcumin in an earler experiment (Figure 3). Overall, these findings indicate that Jak3 activation repress miR-22 expression in malignant T cells. Since the active Jak3 mediates tyrosine phosphorylation and subsequent activation of STAT3 and STAT5 [1-3, 40], we examined whether Jak3-mediated repression of miR-22 was regulated via these transcription factors. Figure 5A shows expression changes in miR-22 (Figure 5A) and STAT3, STAT5A, and STAT5b (Figure 5B) following siRNA-mediated depletion of these STATs in malignant T cells. Inhibition of STAT3, STAT5A, and STAT5B induced a significant increase in the expression of miR22 (Figure 5A) Indicating that Jak3 regulates the expression of miR-22 via both STAT3 and STAT5.

### STAT binding to the miR-22 host gene promoter

**Figure 6:**
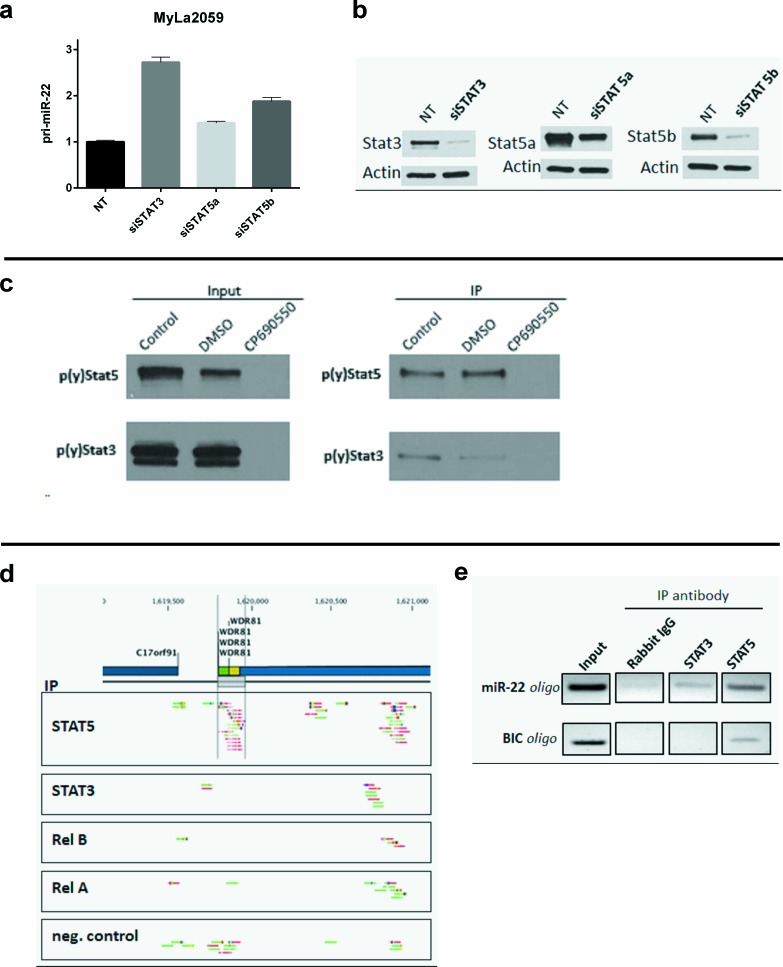
Binding of STAT transcription factors to the the miR-22HG C17orf91 and upstream promoter regions **a.** primary miR-22 expression in MyLa2059 24h following transient transfection with siSTAT3, siSTAT5a, siSTAT5b or non-target (NT) control. Reference GAPDH, error bars reflect variation in technical triplicates. **b.** Knockdown efficiency is evaluated by Western Blot. **c.** Binding of p(y)STAT3 and p(y)STAT5 to an oligonucleotide sequence designed to mimic a putative STAT binding side inside the miR-22HG, C17orf91. Malignant CTCL cell line, Myla2059, was treated for 24h with JAK inhibitor, CP690550 (50μM), DMSO or untreated (control). Protein extracts were subjected to oligonucleotide pulldown and investigated for p(y)STAT3 and p(y)STAT5 presence by Western Blotting before (input) and after (IP) pulldown. **d.** ChIP-seq reads from the C17orf91 promoter region in malignant MyLa2059 cells. Reads obtained from immunoprecipitation with STAT5, STAT3, RelB, RelA and a negative control (Rabbit IgG, bottom). The chromosomal position of C17orf91 and upstream WDR81 refer to hg19. Forward reads are indicated in green and reverse reads are shown in red. **e.** PCR analysis of ChIP samples using the primer set indicated in Supplementary Figure 1. For the C17orf91 promoter the 120bp amplicon was detected in the STAT5- and to a lesser degree the STAT3-precipitated samples. For the BIC (miR-155HG) promoter 190bp amplicon was detected only in the STAT5-precipitated sample.

In addition to their role as transcriptional activators, STAT3 can also function as repressors of transcription. Thus, in some cancer models STAT3 represses the expression of multiple genes including key regulators of stress responses and neoplastic transformation such as p53 [41]. Interestingly, STAT3 has been implicated in both transcriptional activation and repression of the oncomiR-21 [29, 42, 43], which probably reflects a cell-context dependent recruitment of co-activators and co-repressors such as BLIMP-1 [43]. Likewise, STAT5 can function as a transcriptional activator or a transcriptional repressor [44-47]. Accordingly, we examined whether siRNA- mediated depletion of STAT3 and STAT5 influenced the expression of the primary transcript of the miR-22 gene (miR-22HG/C17Orf91), pri-miR-22. As shown in Figure 6A, siRNA-mediated inhibition of STAT3, STAT5A, and STAT5B triggered increased expression of pri-miR-22 suggesting that they - directly or indirectly - repress transcription of miR-22HG.

To elucidate whether STAT3 and STAT5 directly bind to the miR-22HG, we performed oligo-nucleotide-based binding assay using a synthetic oligonucleotide corresponding to a STAT binding sequence in the miR-22HG. As shown in Figure 6C, pY-STAT5 and to a lesser extend pY-STAT3 co-precipitated with the miR-22HG oligonucleotide. Notably, the phosphorylation of STAT3 and STAT5 was almost completely blocked by pre-treatment of malignant T cells with a Jak3 inhibitor, CP-690550, (Figure 6C, left panel). Since this inhibitor blocks STAT3 and STAT5 tyrosine phosphorylation (Figure 6C, left) and transcriptional activity[48], these findings suggest that the activated form of the STAT proteins bind to miR-22HG.

To address further whether STAT proteins directly bind the miR-22HG promoter in malignant T cells, we performed Chromatin Immune-Precipitation followed by DNA sequencing (ChIP-seq) to identify transcriptional targets of STAT and other putative relevant transcription factors in a more physiological setting. ChIP-seq analysis of STAT5-precipitated chromatin from malignant MyLa2059 cells yielded an enrichment of reads comprising a promotor region of the miR-22HG (C17Orf91, Figure 6D). In contrast, only few reads for the miR-22HG promoter were detected in chromatin precipitated with STAT3 antibody (Figure 6D). Since curcumin also inhibits NFκB signaling [49], we also performed chromatin immunoprecipitation with RelA and RelB antibodies. As shown in Figure 6D, precipitation with RelA and RelB antibodies did not yield any reads for the miR-22HG promoter region.

Based on the information obtained from ChIP-seq, we designed primers flanking the STAT-binding site within the promoter (Supplementary Figure S1). These were used in an independent ChIP experiment to detect enrichment by PCR amplification of this sequence in chromatin from malignant MyLa2059 cells precipitated with STAT3 and STAT5 antibodies relative to a negative control antibody (rabbit IgG). Consistent with our earlier experiemental findings, ChIP-seq, PCR amplification (Figure 6E) showed a marked enrichment of the sequence representing the miR-22HG promoter in samples precipitated with STAT5 antibody, whereas STAT3-precipitated samples produced a less pronounced band. In a parallel experiment, we observed enrichment of the sequence representing the STAT5 binding site in the BIC promoter in samples precipitated with STAT5 antibody whereas STAT3-precipitated samples were negative (Figure 6E, lower panel) showing the specificity of the ChIP assay and confirming previous data on a selective STAT5 binding to the BIC promoter [14]. Taken together these data indicate that STAT5, and seemingly to a lesser degree, STAT3 bind to the miR-22HG promoter.

### HDAC inhibitors induce expression of miR-22 in malignant T cells

**Figure 7:**
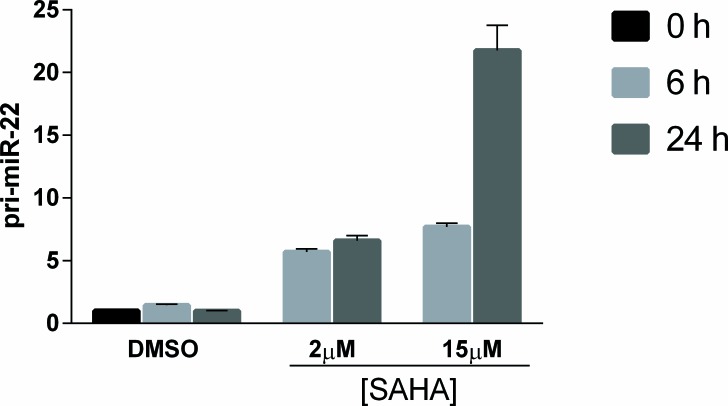
HDAC inhibitor, SAHA, induces time- and concentration-dependent increase in primary miR-22 expression Malignant CTCL cell line, MyLa2059, was treated for 0, 6 or 24 hours with 2μM or 15 μM SAHA or DMSO control. Relative expression of pri-miR-22 was determined by qPCR, reference GAPDH. Error bars reflect variation in technical triplicates.

In addition to a direct transcriptional repression of target genes, STAT3 and STAT5 may also repress transcription through the recruitment of transcriptional co-repressors such as EZH2 and HDACs [44, 46]. Hence, we treated malignant T cells with corresponding inhibitors to address whether repression of pri-miR-22 expression was mediated through these co- repressors. While the inhibitor of EZH2 (DZ-Nep) had no effect on pri-miR-22 expression at concentrations up to 10 μΜ uM (Supplementary Figure S2), a broad HDAC inhibitor, SAHA, used clinically for the treatment of CTCL, triggered a profound, time- and concentration- dependent up-regulation of pri-miR-22 expression (Figure 7). These findings indicate that HDACs play a key role in pri-miR-22 repression, whereas EZH2 most likely do not.

### Elucidating miR-22 targets in malignant T cells

**Figure 8:**
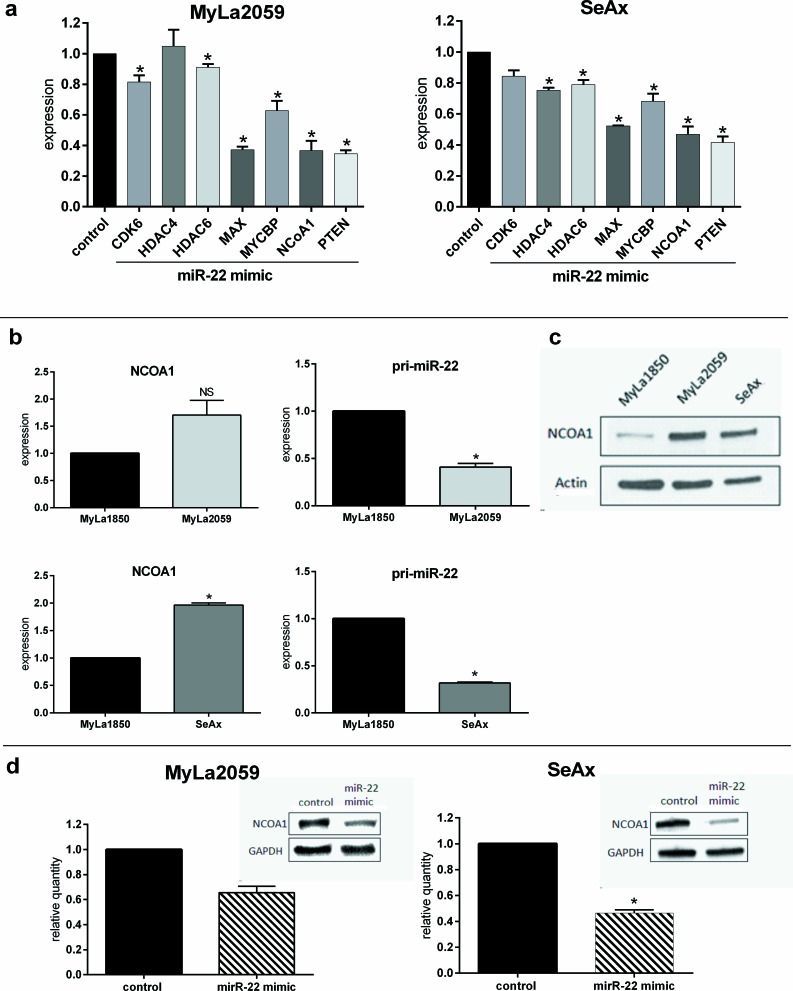
Expression of previously verified miR-22 targets are decreased by miR-22 mimic in malignant CTCL cell lines **a.** Expression of CDK6, HDAC4, HDAC6, MYX, MYCBP, NCoA1 and PTEN following transient transfection with miR-22 mimic relative to a scrambled control in malignant CTCL cell lines (MyLa2059, SeAx) as determined by qPCR. Reference GAPDH, *n* = 7. **b.** qPCR comparison of relative NCoA1-(left panel) and pri-miR-22-(right panel) mRNA expression levels in malignant versus non-malignant cell lines (upper panel: MyLa2059 vs. MyLa1850, lower panel: SeAx vs. MyLa1850). Reference GAPDH, *n* = 3 **c.** Western Blot showing NCoA1 protein expression levels in non-malignant (MyLa1850) and malignant (MyLa2059, SeAx) CTCL cell lines. **d.** Relative expression of NCoA1 protein in malignant CTCL cells (left: MyLa2059, right: SeAx) following transfection with miR-22 mimic or scrambled control. Upper inset is a representative of the Western Blots results for NCoA1 and GAPDH quantification, *n* = 3.

miR-22 has been assigned a role of a tumor suppressor in various solid tumors (reviewed in [38]) as well as some hematological malignancies (acute lymphoblastic leukemia, multiple myeloma and anaplastic large cell lymphoma) [50-53]. While the molecular mechanisms of this miRNA have not been fully elucidated, several miR-22 targets have been identified and validated. Several these targets including HDACs and transcriptional co-activators/regulators of c-Myc [37,39,54,55] could be involved in the pathogenesis of CTCL. To determine whether these proteins were susceptible to inhibition by miR-22 in malignant T cells, we used electroporation to transfect these cells with a synthetic miR-22 (miR-22 mimic). As shown in Figure 8, transfection with a miR-22 mimic inhibited to various degree the expression of all seven verified miR-22 targets examined when compared to transfection with scrambled miR-22 serving as a negative control (Figure 8A). Accordingly, a marked inhibition by 30-60% of MAX, MYCBP, NCoA1, and PTEN was observed in both the malignant T cell lines analyzed: MyLa2059 and SeAx (Figure 8A). Other verified miR-22 targets (CDK6, HDAC4 and HDAC6 [39, 56, 57]) were also inhibited by miR-22 but the effect was much weaker (Figure 8A).

The profound inhibition of NCoA1 by miR-22 is of particular interest as NCoA1 is a transcriptional co-activator regulating multiple oncogenic pathways associated with disease progression and metastasis formation in breast and prostate cancer [34]. When correlating miR-22 and NCoA1 expression in malignant and non-malignant T cells by RT-qPCR (Figure 8B), a relatively high miR-22 expression was associated with a low NCoA1 expression in non-malignant T cells and conversely a relatively low miR-22 expression in malignant T cells was associated with a high expression of NCoA1 mRNA (Figure 8B). These NCoA1 expression levels were also confirmed on a protein level (Figure 8C).

To further substantiate and extend these findings, we transfected malignant T cell lines with a miR-22 mimic and a scrambled control miRNA and measured the miR-22 effect on NCoA1 protein expression. As shown in Figure 8D, miR-22 triggered a significant decrease in NCoA1 protein expression therefore, supporting the notion that miR-22 directly down-regulates the expression of NCoA1 in malignant T cells.

## DISCUSSION

miR-22 has recently been assigned the role of a tumor suppressor miRNA and low expression has been reported in advanced disease and metastasis in several solid cancers (reviewed in [38]). In this work, we provide mechanistic evidence of deficient expression and putative tumor suppressive function of miR-22 in malignant T cells. Our data confirm and extend the recent findings by Ballabio et al. on down-regulation of miR-22 expression in peripheral blood malignant (Sézary) CD4+ T cells [58].

Our experimental molecular studies indicate that the observed low expression of miR-22 in CTCL was due to constitutive repression of miR-22 transcription by aberrant Jak3/STAT signaling in malignant T cells. We document that STAT5 and to a lesser extend STAT3 bind to a STAT-binding motif in the miR-22HG promoter and down-regulate this gene. Thus, inhibition of the Jak3, STAT5A, and STAT5B and STAT3 led to up-regulation of pri-miR-22 and miR-22. We further document that HDAC inhibitors play an important role in regulating miR-22 expression. Most importantly, our results demonstrate that forced expression of miR-22 in malignant cell lines inhibits expression of a number of critical oncogenes including MAX, MYCBP, NCoA1, and CDK6.

It is now well established that in addition to their function as transcription factors, STAT5 and STAT3 can function as transcriptional repressors. For instance STAT5 represses the expression of Cyclin D2 and BCL6 [47, 59], whereas STAT3 represses Glucose-6-Phosphatase and p53 expression [41, 60]. However, relatively little is known about the mechanisms involved in STAT3 and STAT5- mediated repression of gene transcription. Steric hindrance by STAT proteins and recruitment of other co-repressors might account for their function as transcriptional repressors (reviewed in [61]). Thus, STAT5 inhibits expression of the Ig kappa light chain locus by recruiting EZH2, which in turn triggers histone-trimethylation and chromatin conformational changes [42, 44, 62].

Likewise, STAT3 and STAT5 recruit HDACs which, depending on the cellular context, repress or activate transcription through de-acetylation of STAT proteins and other molecules in the transcriptional complexes. An inhibitor of EZHs had no effect on the expression of pri-miR-22, whereas inhibitors of HDACs induced a profound up-regulation in miR-22 expression suggesting that HDACs, but not EZHs, might be involved in the repression of pri-miR-22 transcription.

To assess a putative regulatory function of miR-22 in malignant T cells, we evaluated expression changes of validated miR-22 targets. Specifically, we investigated the effect of miR-22 on the expression of NCoA1, MAX, MYCBP, HDAC4, HDAC6, CDK6, and PTEN, all of which have independently been validated as miR-22 targets [37, 39, 54-57, 63, 64]. Constitutive expression of miR-22 inhibited all of these targets. Four targets (NCoA1, MAX, MYCBP, and PTEN) demonstrated a very high sensitivity to miR-22- mediated inhibition, whereas the other targets were only weakly inhibited by miR-22. The inhibition of MAX and MYCBP was of particular relevance because they have previously been proposed to play a pathogenic role in CTCL due to their effect on the C-Myc oncogene [19, 64, 65].

We, therefore, propose a model where miR-22 expression is low in malignant T cells and is down regulated by IL-2 in normal T cells, while MAX and MYCBP are constitutively active in malignant T cells and are activated by IL-2Rgc cytokines in non-malignant T cells [64]. Therefore, we believe that the repression of miR-22 by IL-2Rgc cytokines and Jak3/STAT activation is not only relevant in relation to CTCL, but might have implications for T cell biology in general.

NCoA1, also known as the steroid receptor co-activator (SRC-1), is a transcriptional co-activator and repressor not previously described in lymphoma but linked to oncogenesis in malignancies such as carcinoma of breast, ovarian, and prostate (reviewed by Walsh [34]). Indeed, global gene repression by NCoA1 promotes oncogenesis and metastatic spread in breast cancer [66,67]. Interestingly, the major clinical importance of an increased expression of NCoA1 in patients with advanced cancer relates to the development of drug resistance [68-71]. For instance, NCoA1 protects cancer cells from tamoxifen induced apoptosis [66,72]. In malignant T cells, as demonstrated in our study, constitutive expression of miR-22 inhibited NCoA1 expression on mRNA and protein levels, confirming its role in regulating NCoA1 expression and function [73]. As NCoA1 regulates retinoid signaling and several other nuclear receptors in addition to the steroid receptor, studies are in progress to address whether NCoA1 plays a role in drug resistance in CTCL and whether co-transfection of miR-22 potentiates the efficacy of anti-cancer therapies and drugs such as bexarotene and tazarotene and other treatment modalities. Indeed, in a study on the efficacy of extracorporeal photochemotherapy (ECP) in CTCL, Berger et al. [74] reported on a dramatic effect on leukocyte gene expression. The study did not include information of miR-22 expression but analysis of the raw microarray data-set obtained from NCBI (GSE23604) (Supplementary Figure 3S (A-C)) indicates that a subset of miR-22 target genes (74 genes) were significantly altered following ECP ( > 2 fold change, *p* < 0.001) (Suppl. Figure S3D). These included validated miR-22 targets (NCoA1 and PTEN) examined in the current study, which were both significantly inhibited post-ECP (Suppl. Figure S3E). These data indicate that NCoA1 and other miR-22 target- genes are inhibited following ECP suggesting a potential role of miR-22 target genes, and by inference restoration of miR-22, in mediating the effect of ECP [74].

Recent data in mice demonstrated that locked nucleotide acid (LNA)-modified oligonucleotides have promising therapeutic potentials and are now tested in a phase one clinical trial [75]. Since LNA-modified oligonucleotides can be designed to mimic both miR-agonists and antagonists, it may be speculated that LNA-based miR-22 mimics might also have a therapeutic potential in cancer treatment.

In conclusion, we demonstrate for the first time, that miR-22 expression is down-regulated in CTCL malignant T cells and propose a molecular mechanism, where aberrant Jak3/STAT signaling leads to STAT3/STAT5 activation and binding to its cognate sequence on the miR-22HG promoter. This binding leads to a direct transcriptional repression of the gene. We further demonstrate that normal function of this tumor suppressor microRNA is to down-regulate a number of putative oncogenes including validated miR-22 targets MAX, MYCBP, HDAC4, HDAC6, CDK6, and NCoA1. Taken together, these findings suggest that Jak3/STAT-mediated repression of miR-22 plays a key role in the pathogenesis and progression of CTCL. Combined with other reports, our results indicate that the Jak3-STAT3/STAT5 pathway may serve as therapeutic targets in CTCL.

## MATERIALS AND METHODS

### Cell lines and culture

Malignant T cell lines, MyLa2059, MyLa2000, PB2B and SeAx were established from patients diagnosed with CTCL [75, 76]. MyLa1850 and MySi are non-malignant T cell lines that were also established from CTCL patients [77]. PSOR2 is a non-malignant cell line established from a patient with psoriasis vulgaris [78].

All cell lines were cultured in RPMI1640 medium (#R2405, Sigma-Aldrich, St. Louis, MO, USA) supplemented with 5% penicillin/streptomycin (Sigma-Aldrich). Furthermore the medium used for MyLa2059, MyLa2000 and PB2B was supplemented with 10% fetal bovine serum (Life Technologies, Roskilde, Denmark). The medium used for SeAx, MyLa1850, MySi and PSOR2 was supplemented with 10% human serum (Blood Bank, State University Hospital, Copenhagen, Denmark) and 10^3^U/mL IL-2 (Proleukin) (Chiron, Emeryville, CA, USA) and 25ng/mL IL-4 (Leinco, St. Louis, MO, USA) (the latter only for PSOR2). Peripheral blood mononuclear cells (PBMCs) were isolated from a patient diagnosed with CTCL as well as from two healthy donors using a Ficoll Gradient, Lymphoprep (Axis-Shield PoC AS, Oslo, Norway) and were described elsewhere [79].

### Inhibitors

JAK inhibitor II (Calbiochem, San Diego, CA, USA); JAK3 inhibitor, CP690550 (Pfizer, Ballerup, Denmark); Curcumin (Alexis, Laufelfigen, Switzerland); SAHA (Vorinostat) (Cayman Chemical, Ann Arbor, MI, USA); Trichostatin A, MS-275 (Entinostat) and Sodium Butyrate (Enzo Life Sciences, Plymouth Meeting, PA, USA); DzNep hydrochloride (Sigma-Aldrich).

### Antibodies

Antibodies used in this work are commercially available and were obtained as follows: NCOA1 antibody from LifeSpan Bioscience (Seattle, WA, USA) (LS-B1702). pySTAT3 antibody from Nanotools (Denzlingen, Germany) (#0036-100/STAT3-9E12). STAT5a and STAT5b antibodies from Santa Cruz Biotechnology (Santa Cruz, CA, USA) (L-20, SC-1081; C-17, sc-835-G). py-STAT5, STAT5, STAT3, RelA, RelB, Histone H3 and rabbit IgG antibodies from Cell Signaling Technologies (Beverly, MA, USA) (#9351, #9358, #4904, #8242, #4922, #4620, #2729). GAPDH antibody from Abcam (Cambridge, UK) (Ab9485) ; and β- Actin antibody from Sigma-Aldrich (A4700).

### Oligonucleotide pulldown assay

Biotinylated oligonucleotides were designed complementary to the predicted STAT binding site (according to the transcription factor search database TFSEARCH version 1.3 http://www.cbrc.jp/papia/howtouse/howtouse_tfsearch.html inside C17orf91/miR-22HG (forward: 5′BIO- TCACTCTTCAGGGAAAAGTGATA-3′; reverse: 5′BIO-TATCACTTTTCCCTGAAGAGTGA- 3′) and were synthesized by Eurofins MWG GmbH (Martinsried, Germany). Lyophilized Ooligonucleotides were resuspended in nuclease-free water and annealed at 95°C for 5 min. Samples were lysed, and the supernatant was pre-cleared with streptavidin agarose beads (Invitrogen, Paisley, UK) for 3h rotating at 4°C. The supernatant was transferred to a new tube and agarose beads as well as 250 pmol oligos per sample were added. Samples were incubated overnight rotating at 4°C. After being washed 5 times with lysis buffer, sample loading buffer was added; the samples were denatured at 100°C for 10 min and subsequently subjected to SDS-PAGE and blotted for pY-STAT3 and pY-STAT5.

Chromatin Immunoprecipitation (ChIP) was performed using SimpleChIP^®^ Enzymatic Chromatin IP kit (Agarose Beads) from Cell Signaling Technologies (Beverly, MA, USA) as previously described [14]. Cross-linked chromatin fragments were captured with antibodies against STAT3, STAT5, RELA, RELB, Histone H3 (positive control) or rabbit IgG (negative control). PCR was performed on immunoprecipitated and control DNA samples using primers for regions upstream of the promoters C17orf91 (miR-22HG) (forward sequence: 5‵TCC TAA AGA GCA GGC GAA AG3‵; reverse sequence: 5‵AAA AAT GCC AAC TCA CAG AGC3‵ ) and BIC (miR-155HG) (forward sequence: 5‵ GAA AGG GAA AGG GGA AAA CA3‵; reverse sequence: 5‵ CGA ACG TGC GAC CCT TTT AT3‵) respectively. Both sets of primers were designed to encompass putative STAT binding sites. PCR annealing temperature was 56°C and amplicon sizes were 120bp and 190bp for miR-22HG- and miR-155HG genes, respectively. The PCR products were separated on a 3% agarose gel using ethidium bromide for visualization.

ChIPseq library construction and sequencing was described in detail in Kopp et al. [14].

### Transient transfections

2*10^6^ cells per sample were transfected with small interfering RNA (siRNA) against JAK3, STAT3, STAT5a, STAT5b or non-targeting control #1 (ON-TARGETplus SMARTpool, Thermo Scientific, Lafayette, CO, USA). Cell pellets were resuspended in 100uL transfection solution (Ingenio Electroporation solution, Mirus Bio, Madison, WI, USA) with 0.5 nmol of the respective siRNAs and transfected with an Amaxa Nucleofector (Amaxa GmbH, Cologne, Germany).

For transient transfection with microRNA mimic (agomiR), 7*10^6^ cells/sample were transfected with 0.25 nmol miR-hsa-miR-22-3p mimic or mimic control (both *miR*Vana^®^ miRNA mimics, Ambion (Sigma-Aldrich, Saint Louis, MO, USA). For SeAx cells a single transfection was performed whereas MyLa2059 cells were subjected to transfections on two consecutive days.

### Protein extraction and western blotting

Protein was extracted from 1*10^6^ cells persample and subjected to SDS-PAGE and Western blotting as described previously [79], [80].

### RNA extraction and qRT-PCR

Total RNA was purified with miR-Neasy Mini Kit (Qiagen, Valencia, CA, USA), cDNA was transcribed from 10ng RNA using TaqMan^®^ miRNA Reverse Transcription Kit (Applied Biosystems, Foster City, CA, USA). Real-time PCR was performed using TaqMan^®^ miRNA assays (Applied Biosystems, Foster City, CA, USA) for miR-22 (#000398) according to the manufacturer's instructions. Expression of U6 (#001973) was used as a reference. For quantification of expression of pri-miR-22 or various mRNAs, RNA extraction was performed with the RNeasy Mini Kit (Qiagen, Valencia, CA, USA). cDNA was subsequently transcribed using the High Capacity cDNA Reverse Transcription Kit followed by PCR analysis using TaqMan^®^ Gene Expression Assays: pri-miR-22 (#Hs03302632_pri), NCOA1 (#Hs00186661), CDK6 (Hs 01026371_m1), HDAC4 (Hs 01041638_m1), HDAC6 (Hs 00195869_m1), MAX (Hs 00811069_g1), MYCBP (Hs 00429315_g1), PTEN (Hs 02621230_s1) (all from Applied Biosystems, Foster City, CA, USA) according to the manufacturer's instructions. GAPDH (#Hs02758991_g1) was included as a reference. The amplification was performed on an Mx3005P qPCR System (Agilent Technologies, Santa Clara, CA, USA) real-time cycler on standard settings.

### Statistics

For statistical analysis a two-tailed Student's *t*-test with a significance level of *p* = 0.05 was used. A significant difference (*p* < 0.05) between a sample and control is indicated with an asterisk.

## SUPPLEMENTARY FIGURES


