# Supplementary material for: Jak3, STAT3, and STAT5 inhibit expression of miR-22, a novel tumor suppressor microRNA, in cutaneous T-Cell lymphoma
Source: Oncotarget. 2015 May 12;6(24):20555–69. doi: 10.18632/oncotarget.4111 (PMC4653025; doi:10.18632/oncotarget.4111)
Supplement: Supplementary file 1 [file oncotarget-06-20555-s001.pdf]

Jak3, STAT3, and STAT5 inhibit expression of miR-22, a novel tumor suppressor microRNA, in cutaneous T-Cell lymphoma

Supplementary Material

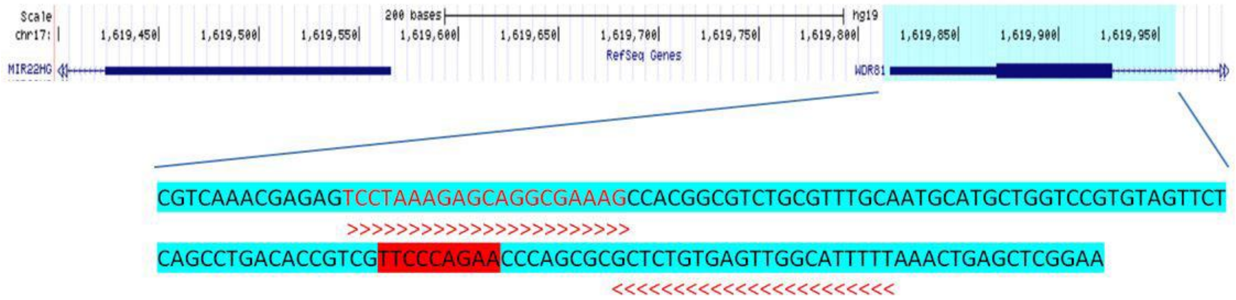

Supplementary Figure S1: Analysis of the C17orf91 (miR-22HG) promoter region providing the most ChIPseq reads (highlighted in turquoise) yielded one STAT binding site (highlighted in red). Primers flanking the binding site that were used for PCR analysis of ChIP samples are indicated in red. The chromosomal position of C17orf91 and upstream WDR81 refer to hg19. For details on the BIC (miR-155HG) promoter region, see [14].

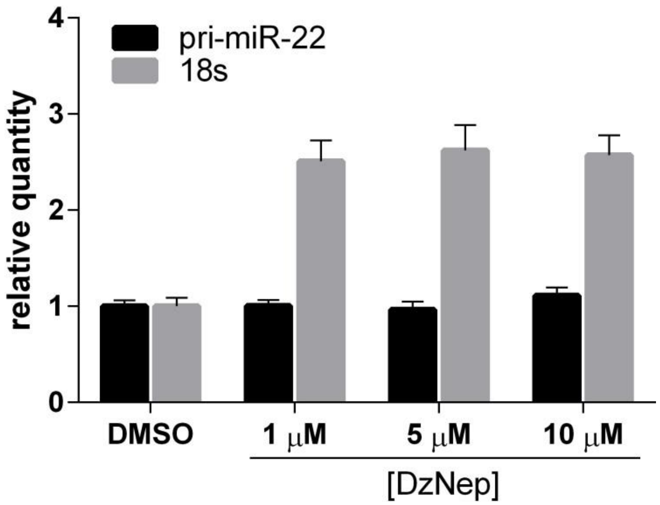

Supplementary Figure S2: DzNep (EZH2) treatment has no effect on pri-miR-22 expression. Malignant CTCL cell line, MyLa2059, was treated for 24 hours with 1, 5 or 10μM DzNep or DMSO control. Relative expression of pri-miR-22 and 18S was determined by qPCR, reference GAPDH. Error bars reflect variation in technical triplicates.

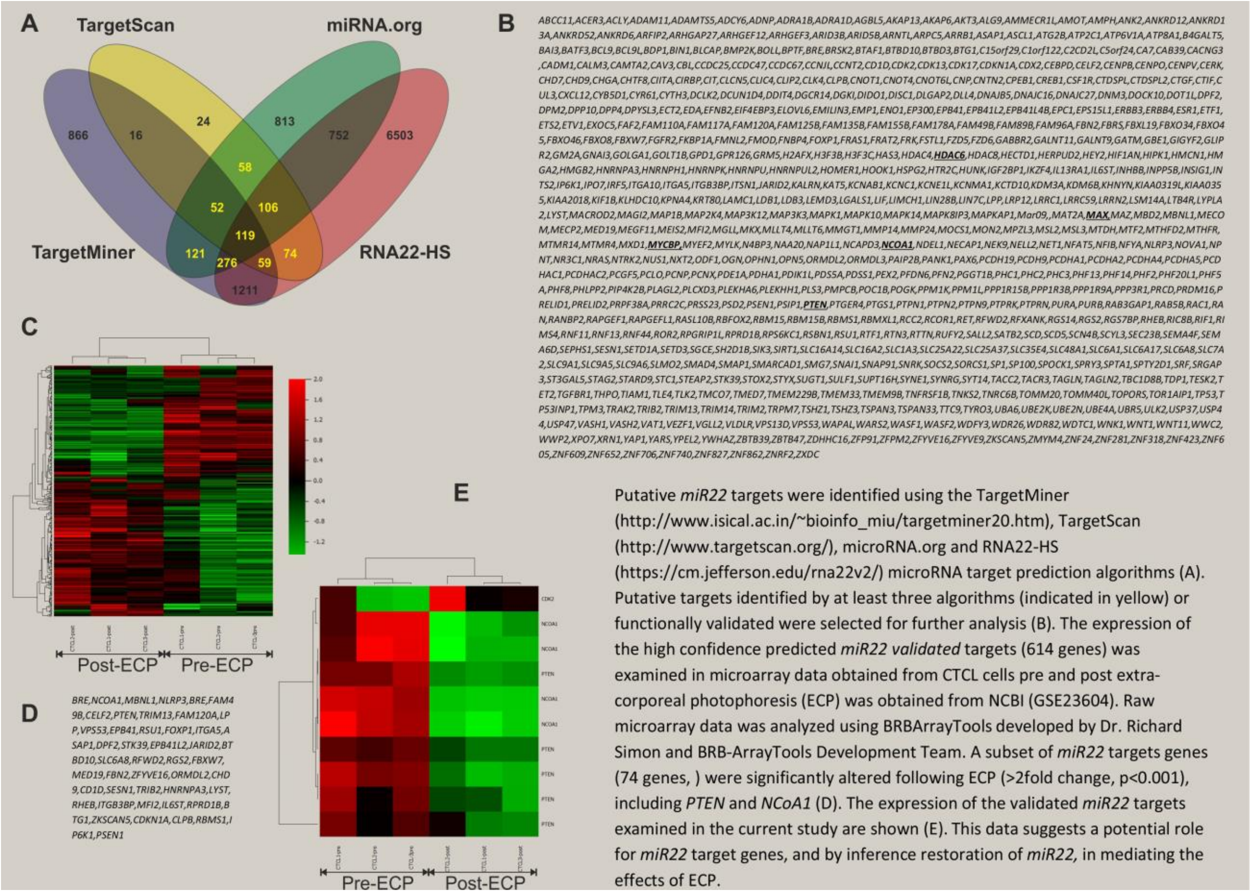

Supplementary Figure S3: Down-regulation of putative miR-22 target genes following extra-corporeal photopherese (ECP). Putative miR-22 target genes were identified using three miRNA target prediction algorithms as indicated (A).Putative targets identified by at least three algoritms (indicated in yellow) or functionally validated were selected for further analysis (B). The expression of the high confidence predicted miR-22 validated targets (614 genes) was examined in microarray data from cells derived from CTCL patients pre- and post- ECP obtained from NCBI (GSE23604)[74]. Raw microarray data was analysed using the BRBArrayTool developed by Dr. Richard and the BRB-ArrayTools Developments Team. A subset of miR-22 target genes (74 genes) were significantly altered following ECP (<2 fold change, p< 0.001), including NCoA1 and PTEN (C and D). The expression of the validated miR-22 targets examined in the current study are shown (E).
